# Cell Type Classification and Unsupervised Morphological Phenotyping From Low-Resolution Images Using Deep Learning

**DOI:** 10.1038/s41598-019-50010-9

**Published:** 2019-09-17

**Authors:** Kai Yao, Nash D. Rochman, Sean X. Sun

**Affiliations:** 1 Department of Mechanical Engineering, Johns Hopkins University, Baltimore, Maryland United States of America; 2 Institute for NanoBioTechnology, Johns Hopkins University, Baltimore, Maryland United States of America; 3 Physical Sciences in Oncology Center, Johns Hopkins University, Baltimore, Maryland United States of America

**Keywords:** Computational models, Classification and taxonomy

## Abstract

Convolutional neural networks (ConvNets) have proven to be successful in both the classification and semantic segmentation of cell images. Here we establish a method for cell type classification utilizing images taken with a benchtop microscope directly from cell culture flasks, eliminating the need for a dedicated imaging platform. Significant flask-to-flask morphological heterogeneity was discovered and overcome to support network generalization to novel data. Cell density was found to be a prominent source of heterogeneity even when cells are not in contact. For the same cell types, expert classification was poor for single-cell images and better for multi-cell images, suggesting experts rely on the identification of characteristic phenotypes within subsets of each population. We also introduce Self-Label Clustering (SLC), an unsupervised clustering method relying on feature extraction from the hidden layers of a ConvNet, capable of cellular morphological phenotyping. This clustering approach is able to identify distinct morphological phenotypes within a cell type, some of which are observed to be cell density dependent. Finally, our cell classification algorithm was able to accurately identify cells in mixed populations, showing that ConvNet cell type classification can be a label-free alternative to traditional cell sorting and identification.

## Introduction

Convolutional neural networks (ConvNets), a main component in many deep learning algorithms, have been applied to a strikingly broad array of tasks in image processing. From stylistic transfer in artwork^1^ to medical image analysis^2–5^, ConvNets have been trained to successfully generate, segment, and classify different types of images, often outperforming experts (conducting manual processing) across various fields. The application of these tools to cell type identification is a topic of growing interest owing to the fact that traditional techniques require complex laboratory procedures including antibody staining or RNA/DNA identification. Cell type classification is often performed using flow cytometry^6^ which requires suspending the cells, altering cell morphology, and potentially changing the proteome. Worse, cell fixation is commonly required to carry out these protocols irrevocably altering the cell from its native state. Accurate classification protocols of live mammalian tissue cells in mixed populations requiring only transmitted light images are particularly sought after for the study of stem cell differentiation dynamics^7^ which are especially sensitive to fluorescent labeling. Motivated by the broad success of ConvNets across fields and the man possible applications in cell biology, work demonstrating the successful segmentation^2^ and classification^3–5^ of single adherent cells as well as mixed populations in clusters and in suspension has begun to gain traction. However, hurdles still persist regarding the application of ConvNets to single cell classification.

First, there is a wide variety of possible morphological phenotypes among mammalian cells, and qualitative morphological differences may be observed across different conditions, or experiments conducted within days of one another. These morphological variations are great enough to preclude generalization of the network to novel data even when validation within the original dataset is near perfect (see Results).λSecond, most published methods rely on high resolution images captured through objectives with large numerical apertures (NA).^8^ These high NA lenses have very short working distances requiring cells to be plated on thin glass coverslips before imaging which increases the number of experimental steps, time spent at the bench, and ultimately cost to the lab. To address these issues, we propose a label-free classification scheme using typical thick-bottomed, plastic cell culture flasks, in which live cells are most commonly maintained in cell biology laboratories, allowing for a larger amount of data to be collected with less expense.

Here we establish a method for cell type classification utilizing only brightfield images taken on a benchtop microscope directly from cell-culture flasks. Despite the low resolution of the images obtained and significant flask-to-flask heterogeneity, we are able to demonstrate both high accuracy and generalizability to novel data. To further measure the predictive value of the network, we recruited 20 individuals with cell culture experience to perform visual inspection and manual classification, so called “expert classification” (see Methods and Materials). Notably, when given images containing multi-cell regions, experts performed well despite poorly classifying single cells. This finding suggests experts rely on the identification of characteristic phenotypes within subsets of each population instead of ubiquitous identifiers.

To uncover potential biological significance of these morphological phenotypes, we developed Self-Label Clustering (SLC), a novel unsupervised clustering method designed to cluster and identify distinct morphological phenotypes within a single cell type. Single cells undergo significant morphological changes throughout the cell cycle, and even clonal populations span a wide range of single cell morphologies which may benefit from a larger vocabulary of identifiers than currently available. Using SLC, we are able to perform clustering of single cell morphologies from low-res images taken in cell culture flasks and describe cell density-dependent phenotypes. This result highlights the use of unsupervised methods in machine learning (ML) to establish novel morphological identifiers. Moreover, our SLC algorithm is able to demonstrate a dependence on cell density regarding the exploration of this phenotypic space, indirectly suggesting the presence of diffusible factors contributing to cell-cell communication, and leading to a measurable impact on cell shape. ML techniques are commonly applied towards problems in data analysis, and we believe detailed examination of the hidden features resulting from ConvNet training, as highlighted in this method, will aid in theory and model development for biological systems as well. As increasingly advanced experimental techniques yield ever-deepening data sets, there will be an even greater need for theoretical frameworks capable of explaining complex observations^9^.

## Results

### Single cell morphological heterogeneity

**Figure 1:**
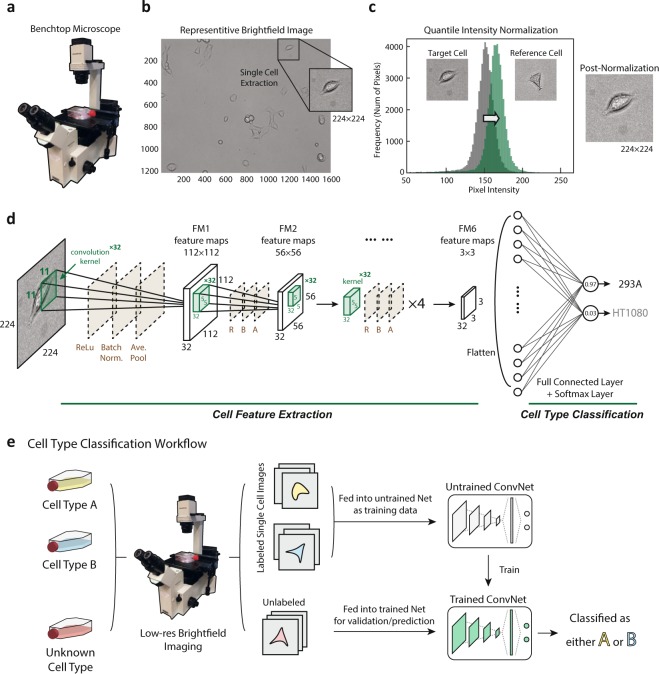
Data preparation, workflow, and ConvNet architecture. (**a**) A benchtop microscope (model OLYMPUS IX50) was used to capture cell images in commonly-used, thick bottomed, plastic cell culture flasks. (**b**) Example brightfield images of both multi-cell regions (1216 × 1616 pixels) and manually cropped single cell regions (224 × 224 pixels) are shown. Note the camera used was not monochromatic and the images were converted to grayscale to reduce dimensionality and improve training efficiency. (**c**) Quantile normalization of the image intensity distribution was performed for each cell to a reference distribution constructed from an arbitrarily selected single cell image. (**d**) Illustration of the proposed ConvNet architecture: Six quadruplets of convolutional, ReLU, Batch Normalization and average pooling layers were constructed. A single fully connected layer was constructed before the Softmax and classification layers. Six convolutional activations (feature maps) after being average-pooled, notated’FM1’ through’FM6’. (**e**) Illustration of the Cell Type Classification workflow from low-res flask images to the artificial neural network. Labelled cells (yellow, blue) in labelled flasks were given to the neural network as training data, and unlabeled flasks were treated as validation or test data. The trained ConvNet model is able to predict the cell type with low error given novel cells with unknown labels.

In the present study, we establish a cell type classification pipeline designed for use by cell biology groups utilizing low-resolution images and requiring minimal additional effort from the experimentalist beyond a traditional workflow for cell passage. A benchtop microscope was used for imaging directly from cell culture flasks (Fig. 1a, see Materials and Methods). Low-res brightfield images were recorded using the standard microscope software (Fig. 1b, see Materials and Methods). The larger field of view was then cropped into single cell images of size 224 × 224 for network training input (Fig. 1b) after normalization (Fig. 1c). Figure 1d shows the cell classification ConvNet architecture: with the cell image input layer, six quadruplets of convolutional, ReLU, Batch Normalization and average pooling layers, a fully-connected layer and a softmax classification layer (see Materials and Methods). Detailed neural network structure is displayed in Table 1. After training, the ConvNet is capable of classifying cell type with low error (Fig. 1e). This workflow is illustrated in (Fig. 1e).

**Table 1:** Cell type classification ConvNet structure.

Layer Type	Learnable Parameters	Activations
Image Input	—	224 × 224 × 1
Convolution	Weights: 11 × 11 × 1 × 32	224 × 224 × 32
	Bias 1 × 1 × 32	
Batch Normalization	Offset: 1 × 1 × 32	224 × 224 × 32
	Scale: 1 × 1 × 32	
ReLU	—	224 × 224 × 32
Average Pooling	—	112 × 112 × 32
Convolution	Weights: 5 × 5 × 32 × 32	112 × 112 × 32
	Bias 1 × 1 × 32	
Batch Normalization	Offset: 1 × 1 × 32	112 × 112 × 32
	Scale: 1 × 1 × 32	
ReLU	—	112 × 112 × 32
Average Pooling	—	56 × 56 × 32
Convolution	Weights: 5 × 5 × 32 × 32	56 × 56 × 32
	Bias 1 × 1 × 32	
Batch Normalization	Offset: 1 × 1 × 32	56 × 56 × 32
	Scale: 1 × 1 × 32	
ReLU	—	56 × 56 × 32
Average Pooling	—	28 × 28 × 32
Convolution	Weights: 5 × 5 × 32 × 32	28 × 28 × 32
	Bias 1 × 1 × 32	
Batch Normalization	Offset: 1 × 1 × 32	28 × 28 × 32
	Scale: 1 × 1 × 32	
ReLU	—	28 × 28 × 32
Average Pooling	—	14 × 14 × 32
Convolution	Weights: 5 × 5 × 32 × 32	14 × 14 × 32
	Bias 1 × 1 × 32	
Batch Normalization	Offset: 1 × 1 × 32	14 × 14 × 32
	Scale: 1 × 1 × 32	
ReLU	—	14 × 14 × 32
Average Pooling	—	7 × 7 × 32
Convolution	Weights: 5 × 5 × 32 × 32	7 × 7 × 32
	Bias 1 × 1 × 32	
Batch Normalization	Offset: 1 × 1 × 32	7 × 7 × 32
	Scale: 1 × 1 × 32	
ReLU	—	7 × 7 × 32
Average Pooling	—	3 × 3 × 32 (LCA*)
Fully Connected	Weights: 2 × 288	
	Bias 2 × 1	1 × 1 × 2
Softmax	—	1 × 1 × 2
Classification Output	—	—

*LCA = Last Convolutional Activations (see Self-Label Clustering).

**Figure 2:**
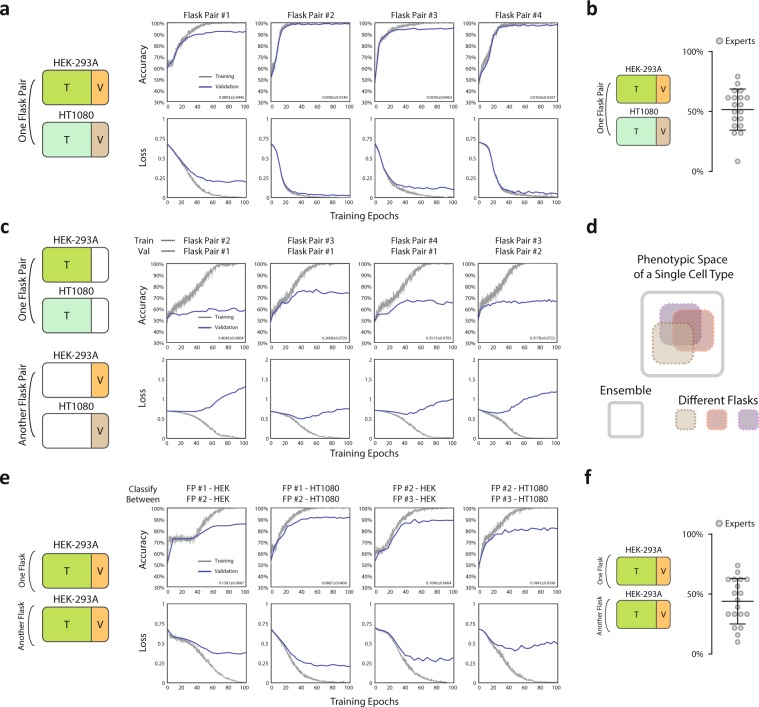
Within-flask validation shows excellent performance while cross-flask generalization is not achieved. a. Left: Illustration of training and validation dataset in this paradigm (within-flask). Right: Accuracy and loss for both training and validation across training epochs. Validation of over 95% accuracy can generally be achieved. (Number of cells: Flask Pair #1 - 272 (HEK-293A), 441 (HT-1080); Flask Pair #2 - 305 (HEK-293A), 497 (HT-1080); Flask Pair #3 – 120 (HEK-293A), 307 (HT-1080); Flask Pair #4 - 208 (HEK-293A), 219 (HT-1080)). Right-bottom corner of each accuracy panel: 95% confidence interval of classification error. (**b**) Expert classification results corresponding to panel (a) of within-flask pair validation. (**c**) Cross-flask pair validation shows poor model performance by trained neural network. Left: Illustration of training and validation dataset in this test, Right: Accuracy and loss for both training and validation across neural network training epochs. Validation of over 70% accuracy is generally not achieved. Right-bottom corner of each accuracy panel: 95% confidence interval of classification error. (**d**) Illustration of the proposed distribution of single cell morphological phenotypes across different flasks. (**e**) Classification of the same cell type in two separate flasks shows excellent performance indicating flask-to-flask single cell heterogeneity in morphological phenotypes. Left: Illustration of training and validation dataset in this test, Right: Accuracy and loss for both training and validation across neural network training epochs. Validation of over 95% accuracy can generally be achieved. Right-bottom corner of each accuracy panel: 95% confidence interval of classification error. (**f**) Results for expert classification between two flasks (panel e) of the same cell type.

We initially trained and validated the network on single cell images originating from a pair of flasks in a single experiment, i.e. images from one flask of cell type A (Human embryonic kidney cell line HEK-293A) were labelled positive and images from another flask of cell type B (Human fibrosarcoma tumor cell line HT1080) were labelled negative. Two flasks from one experiment are considered as one’flask pair’ illustrated in Fig. 2a (left). Randomly assigning 80% of the cells as training data and 20% as validation, for every flask pair so tested, we were able to achieve satisfactory validation accuracies in excess of 95% (Fig. 2a) averaging over mini-batches, with comparable training accuracies well above expert classification with an average of 51.6% (Fig. 2b). To ensure the expert classification was a direct comparison of this training regime, all cells came from one flask pair of HEKs and HT1080s (Fig. S3). The computing time spent on the ConvNet training for each trial was fairly short generally requiring less than 5 minutes to achieve over 95% validation accuracy (NVIDIA GeForce GTX 1060 6GB), suggesting the method we developed is easy, accurate and fast to apply. Convolutional activations (unpooled feature maps) for representative cells of the two cell types are shown in (Fig. S2) for six convolutional layers respectively.

**Figure 3:**
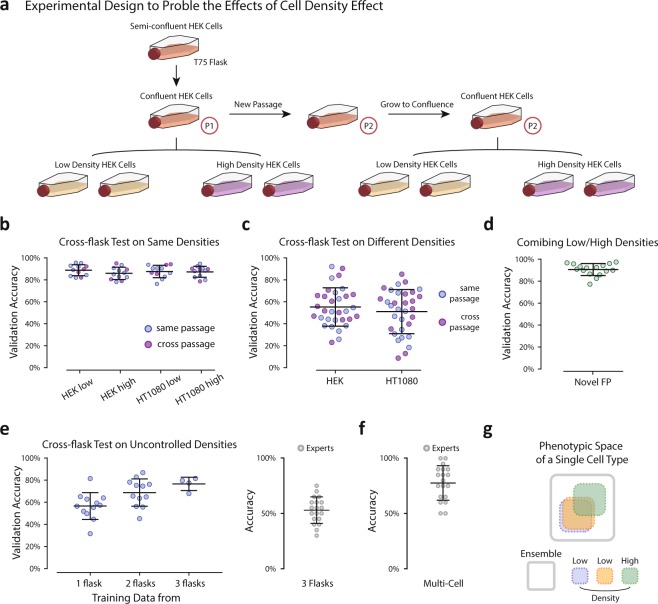
Single cell seeding density significantly affects cell morphology. (**a**) Illustration of the experimental design to probe the effects of cell density across flasks. (Number of cells: HEK-293A low density 326 (P1), 352 (P2); high density 312 (P1), 313 (P2); HT1080 low density 507 (P1), 621 (P2); high density 556 (P1), 504 (P2)). (**b**) Cross-flask tests with conserved densities (e.g. high density training/high density validation, low density training/low density validation). Tests for which the training and validation data were both from the same passage were labeled blue and tests for which the training and validation data were from different passages were labeled purple. There was no observed dependence on passage number. (**c**) Cross-flask tests across densities (e.g. high density training/low density validation, low density training/high density validation) and colored according to passage as in (**b**). (**d**) Training on both densities and generalizing to a novel flask pair with uncontrolled density. (**e**) Left: Training on pools of images from flask pairs of uncontrolled density and generating to novel flask pairs of uncontrolled density. Right: expert classification training on 3 uncontrolled flask pairs and generalizing to a novel flask pair of uncontrolled density. (**f**) Expert classification for multi-cell frame classification of two cell types. (**g**) Illustration of the proposed distribution of single cell morphological phenotypes within a single cell type across flasks of different densities.

**Figure 4:**
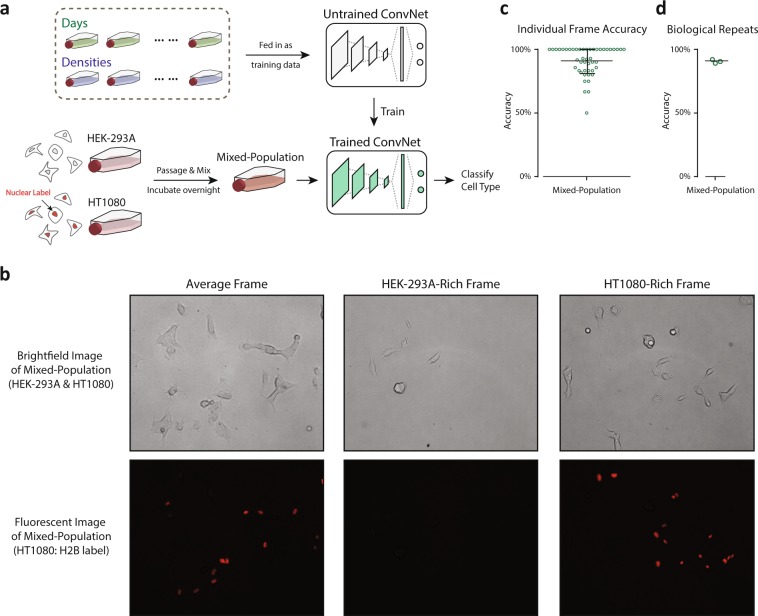
ConvNet accurately achieves mixed-cell population identification. (**a**) Mixed-cell experiment preparation and application workflow. Two types of cell were passaged and mixed together before being incubated overnight. The mixed-cell population was then identified through the same protocol as illustrated in Fig. 1e for cell type classification. Data from different days (uncontrolled densities) and controlled densities were pooled together for ConvNet training. (**b**) Representative brightfield images and fluorescent images of mixed-cell population of HEK-293A and HT1080.Three representative pairs are shown, one frame with similar number of HEK-293A and HT1080 cells, one HEK-293A-rich frame, and one HT1080-rich frame. (**c**) Accuracy of individual frames, identifying cell types from mixed-cell population (from one biological repeat). (**d**) Accuracy of 3 biological repeats of mixed-cell population classification.

We proceeded to test the generalizability of the network, and asked whether training from one pair of flasks (one containing cell type A and the other containing cell type B) are generalizable to a different pair of flasks grown on a different day. We took images from 4 pairs of flasks (one containing A and the other containing B cells). Each pair of flasks contained cells of a different passage number grown on a different day. From this data, any pair of the flasks can be used as a training data set, and the rest of the pairs can be used for validation. To maintain consistency, the training options (network hyperparameters) were kept the same in all trained networks (including the training-validation ratio of 4 to 1), and was the same as single flask pair analysis in (Fig. 2a). In total this amounted to 4 sets of training data and 12 sets of validation data. Despite ideal training accuracy approaching 100% for all training data, we were surprised to find validation on a different flask pair on a different day was poor and only slightly better than random (Figs 2c and 4 of 12 possible pairs shown). This suggests any single flask on a single day only spans part of a much larger morphological space. We went on to perform the following cross-flask exercise in an effort to utilize a more robust training set. Cells from multiple pairs of flasks were pooled as training data with the remaining pairs left for validation: e.g. 1, 2, and 3 pairs of flasks can be used as training data, and pair 4 can be used for validation. This exercise yielded 12 (4choose1*3choose1), 12 (4choose2*2choose1) and 4 (4choose3*1choose1) validation data points respectively. Again training options were maintained. We found that the average accuracy increased as the pool size of the training set increased, indicating day-to-day, flask-to-flask heterogeneity, while great enough to prohibit generalization from a single day of imaging, does not prohibit generalization from a larger pool of training data (Figs 2e and 3e).

Given the identification of significant heterogeneity between flasks of the same cell type imaged on separate days, we went on to determine if the network was capable of identifying flasks of the same cell type from separate pairs of data taken on different days (illustrated in Fig. 2e, left). Indeed the network was able to accurately identify the same type of cells from different flasks well above expert classification (Fig. 2e,f), performing no worse than the HEK vs HT1080 classification shown in (Fig. 2a). These results (Fig. 2a,c,e) were all obtained from similarly prepared flasks at single cell density and imaged under the same conditions. The only unknowns are the exact number of cells seeded, imaging day-dependent biological variables (e.g. passage number), and the intrinsic variability of the imaging protocol (ambient light etc.). These unknowns are often dismissed as irrelevant during routine cell seeding, but here we discover that the morphological spaces occupied by cells from the same cell type but different flasks were sufficiently different to affect the outcome of the model learned by the network. This result suggests that each flask only occupies a portion of the morphological space and the ConvNet is able to distinguish them (Fig. 2d). The construction of larger data sets through the pooling of multiple flasks may be shown to overcome this problem (see Fig. 3d,e).

### Mixing cell densities achieves robust generalization

Cell density is known to affect single cell phenotype specification including the regulation of cell growth via various mechanisms of cell-cell interaction^10–12^, and cell morphology^13,14^. In all of the flasks prepared for this study, the density was well within the single-cell limit (no appreciable physical cell-cell contact); however, the failure of the ConvNet to generalize between flasks (Fig. 2c) and the observation that the network is able to distinguish different flasks of the same cell type (Fig. 2e) motivated us to design new experiments for which the number of cells seeded into each flask was precisely controlled. We selected two seeding densities, “high” with 0.5 million cells seeded per flask and “low” with 0.1 million cells seeded per flask (Fig. 3a). To ensure differences in cell density were the cause of the observed network performance, and not another source of biological variation (e.g. passage number) or the intrinsic variability of the imaging protocol, we established the following protocol (Fig. 3a).

On day one, for each cell type, a T75 cell culture flask was prepared and incubated for 24 hrs after which time it was split into four flasks, 2 of high density and 2 of low density. The remaining cells from the flask were seeded in a new flask. The four density-controlled flasks were allowed to adhere for 24 hrs and were imaged within 2 hrs of one another. The new flask was maintained for one week during which time it was passaged once before the process of splitting into density-controlled flasks was repeated. In total this produced 16 flasks: 2 low density and 2 high density for each cell type imaged on two separate days from two separate passages.

We found that flasks of the same density were able to generalize to one another regardless of the day on which they were imaged, i.e. neither the passage number (Fig. 3b) nor any other imaging-specific variable impedes generalization. However, networks trained on images of one density were not able to generalize to flasks of the other (Fig. 3c). While these results clarified that variations in cell density, even within the single-cell regime, were the cause of this inability to generalize the network model to novel data, they highlighted a problem in the application of this protocol for everyday lab use. We constructed the method presented in (Fig. 1) to require no dedicated imaging, and only used images captured within cell culture flasks so that cell passage would not be required prior to classification. If only small variations in cell density preclude generalization of the model, then inherent heterogeneity within cell culture flasks may make this approach impractical. However, we found generalization to novel data of either density is still possible when the network model is trained on a combination of both densities (Fig. 3d). We went on to assemble combinations of flasks of uncontrolled cell density (discussed in the previous section) and found that, indeed, utilizing pairs or triplets of flasks for training data succeeded in increasing the validation accuracy up to 80% (Fig. 3e, left), well beyond expert classification (Fig. 3e, right). Thus, the method presented is amenable to integration within the typical work-flow of a cell biology lab without the need for dedicated imaging or cell passage prior to data collection. The only additional requirement is that a few independent flasks of each cell type of interest should be pooled as training data to achieve better generalization.

### Expert classification

The morphological differences between flasks of uncontrolled density were not great enough to achieve expert classification (Fig. 2f). This was unsurprising considering expert performance distinguishing between two different cell types was only slightly better than random (Fig. 2b); however, expert classification of images containing multiple cells was better (Fig. 3f). Images containing multi-cell regions (1216 × 1616 pixels) were displayed to experts in the manner described above for single cell images. In this task, experts showed good performance, achieving 77% accuracy on average. This result suggests experts rely on the identification of characteristic phenotypes within subsets of each population of cells instead of ubiquitous identifiers of each single cell. In other words, experts do not identify cell type based on a set of characteristics shared by the majority of cells of one cell type and absent from the majority of the other, but rather through the identification of characteristic phenotypes present in a few cells out of the group or perhaps the relative frequencies at which these minority phenotypes are observed.

### ConvNet can achieve classification in mixed cell populations

One of the ultimate utilities of the present work is to distinguish cell types from a mixed population of cells of unknown origin. This often occurs during primary cell extraction and isolation where after introducing samples into flasks, there is growth of a mixed population of cells. Typically, those cells are then sorted using an established molecular label, or by morphological features such as size where extra input is required. Flow cytometry^6^ using fluorescent markers has been widely applied in cell sorting to create sub-populations. However, trypsinization and detachment of cells are required to carry out flow cytometry, and a majority of cells are often discarded in the experiment, leading to low sensitivity (true positives/(true positives + false negatives)) even though specificity (true negatives/(true negatives + false positives)) may be quite high. We asked whether our ConvNet architecture is able to distinguish cells in a mixed cell population grown together over night in the same flask. To demonstrate this, we utilized the expression of a H2B-mCherry label in the nuclei of HT1080 cells, which can be identified in the fluorescence channel, and unlabeled HEK-293A cells. The detailed experimental design is described in (Fig. 4a) as well as the Materials and Methods. We captured brightfield images (as described in Fig. 1) of the mixed-cell population (Fig. 4b) and pooled all flasks of the two cell types including density uncontrolled and controlled flasks as training data for the cell type identification. The ConvNet showed excellent performance (Fig. 4c) and was able to achieve over 90% accuracy when trained by pooling all flasks in (Figs 2 and 3) at all controlled and uncontrolled cell densities, indicating both high sensitivity and specificity. Three biological repeats were performed and the accuracy is reported in (Fig. 4d). As (Fig. 4b) shows, frames (photos) with both even distributions of the two cell types as well as uneven distributions of the two cell types (HEK-293A-Rich or HT1080-Rich) were observed. This corresponds to different regions in the mixed-cell population flask: some regions with more HEK-293A cells and some with more HT1080 cells. The classification quality of individual frames (Fig. 4c) indicates that even though in the mixed-cell population flask there exist regions with uneven cell type distributions, with the Machine Learning model most frames can be classified with high accuracy and the average accuracy over all frames is over 90%, showing excellent generalization when pulling data from multiple flasks.

### Self-label clustering: unsupervised morphological phenotype identification

Having established the likely presence of distinct morphological phenotypes within each cell type, we sought to determine if these phenotypes can be identified and distinguished computationally. Several unsupervised clustering techniques were tried including Principle Component Analysis (PCA)^15^. PCA, widely applied in facial recognition^16^, is a numerical dimensionality-reduction technique which extracts the dimensions of a dataset with the largest possible variances. *k*-means and *k*-medoids algorithms are then often applied to provide clusters in a lower dimensional subspace.

We found PCA to be poorly suited for this application with clusters identified by cell orientation (relative to the camera lab-frame and of no biological significance) dominating the results (see Fig. S4, cluster #1 contains cells oriented north-west to south-east, and cluster #2 contains cells oriented north-east to south-west).

**Figure 5:**
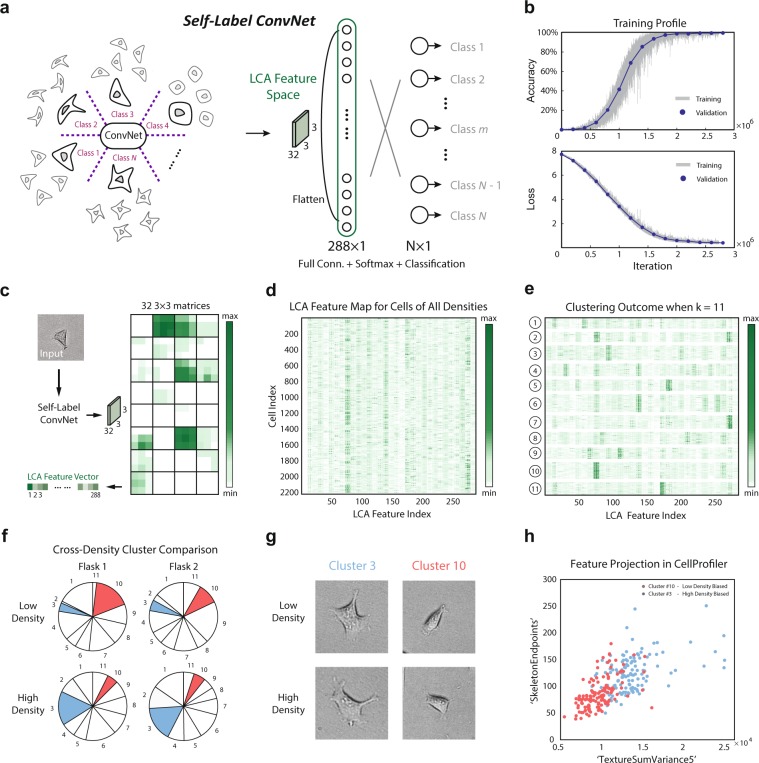
Self-Label Clustering is able to identify distinct morphological phenotypes within a single cell type. (**a**) Illustration of the Self-Label ConvNet architecture. The group of augmented copies for each cell are considered unique classes, yielding the same number of classes in the final layer as there are cells used to train the network. The [l]ast [c]onvolutional [a]ctivation or’LCA’ feature space, labeled in green, is the structure of interest for the following morphological phenotype clustering. (**b**) Training profile of Self-Label ConvNet. An accuracy of nearly 100% can be achieved for both training data and validation data, and a Softmax loss of nearly 0 can be achieved for both training data and validation data. (**c**) Workflow for acquiring the LCA Feature Space for an example cell. Novel cells are input into the pre-trained Self-Label ConvNet and the activations of the last convolutional layer are recorded as 32 3 × 3 matrices for each cell input. The matrices are then flattened to a vector of length 288, each element representing one’feature’ of the input cell. (**d**) LCA matrix: LCA Feature Maps for many cells across all densities (2208 cells total) were displayed as rows in a matrix (size 2208 × 288) with each column representing one feature in the LCA. (**e**) Clustering outcome for the LCA matrix applying *k*-means to rows according to Euclidean distance with *k* = 11. Clusters were shown after reshuffling the cell indices based on their cluster index.. (**f**) Cross-density cluster comparison. Two flasks of two densities were shown. For each flask, the fraction of cells belonging to each of the *k* = 11 clusters was displayed. Clusters with significantly different representations between densities were colored. (**g**) Representative cell images of the clusters #3 and #10 for two densities of cells. (**h**) Morphological Analysis: Two clusters of cells dominated by low density (cluster #10) and high density (cluster #3) respectively, were analyzed. Morphological properties for cells within these clusters were calculated with CellProfiler, and two features (SkeletonEndpoints and TextureSumVariance5) were chosen to generate a 2D projection, illustrating clear distinguishability in a low dimensional morphological feature space. High density biased cluster (cluster #3) was labeled in red and low density biased cluster (cluster #10) was labeled in blue.

We sought to construct a novel, unsupervised clustering technique to uncover morphological identifiers of potential biological significance. For this section of the work we used images of only cell type A (HEK-293A). We began by constructing”self-classes” for each cell, each class representing a series of (50) images generated out of the same cell through the augmentation outlined in (Fig. S1a). We assigned each class a unique label and replaced the final two-class classification layer in the network architecture described in (Fig. 1d) with an *N* -class classification layer where *N* is the number of classes determined by the number of cells in the database (Fig. 5a). In this way, we constructed what we call a Self-Label ConvNet where the groups of augmentations of each cell are considered unique classes. When given each original image used to generate these classes, the trained Self-Label ConvNet model is able to return a representation of the similarities and differences among any group of the original images based on learned features present in the hidden layers of the network. These similarities and differences are in the vocabulary of novel features learned by the network training without relying on any predetermined set of morphological identifiers.

All cells analyzed from every density-controlled flask were included in the training data of Self-Label ConvNet in order to span the entire morphological feature space of HEK cells. We then trained the network on 80% of the data and validated on the remainder achieving close to 100% both training accuracy and validation accuracy (Fig. 5b). The ‘original’ cell augmented to generate each class was excluded from both the training and validation sets. These cells were then classified through the trained Self-Label ConvNet and the activations of the last convolutional layer were recorded after the final average-pooling (32 3 × 3 matrices). The matrices were then flattened to a vector of length 288, each element representing one ‘feature’ of the input cell (Fig. 5c).

This feature space vector of size 288 × 1 is composed of the [l]ast [c]onvolutional layer’s [a]ctivations, which we call the “LCA Feature Space”. A matrix with rows containing LCA vectors for each cell was displayed in (Fig. 5d). *k*-means clustering was then performed on the rows of this matrix in (Fig. 5d) using Euclidean distance. Features learned during ConvNet training have been successfully utilized for clustering before^17^ and unsupervised clustering of images based on their ConvNet representations has recently been shown to successfully recognize objects within the ImageNet database^17^. The Self-Label ConvNet described in this study similarly recognizes sub-classes within a single cell type without prior knowledge of these classes. We first showed that the proposed unsupervised clustering approach is robust to cell image augmentations through examining the LCA Feature Space of a single cell with its augmentations and found that the LCA difference between augmentations is minor (Fig. S5a). Cluster numbers from 2 to 100 were then tested and a peak silhouette score (Fig. S5b, top) was observed for *k* = 11 where the clustering error indicated by the sum of the distances to cluster centers also appears to change slope (so-called”elbow plot” in (Fig. S5b), bottom).

The LCA matrix with rows reordered based on cluster identity is displayed in (Fig. 5e) and the distances between pairs of LCA Feature Spaces (pairs of cells) are displayed in (Fig. S5c) with the low distances on the diagonal indicating cells are similar to other cells belonging to the same cluster. The clusters identified through the application of the Self-Label ConvNet were then examined for the presence of any density dependence.

The fractions of the populations lying in each cluster were displayed for two low and two high density flasks in (Fig. 5f). The colored clusters are of special interest being relatively enriched or depleted in one density compared to the other. Approximately 16.25% of cells in high density flasks belong to cluster A (#3, red) while only 4.56% of cells at low density reside in cluster A. Similarly, approximately 13.44% of cells in low density flasks belong to cluster B (#10, green) while only 5.36% of cells at high density reside in cluster B. The representative cell images of the two clusters in low and high densities are shown in (Fig. 5g) and also in (Fig. S5d). Cells from clusters A and B were collected and analyzed through the CellProfiler platform to determine any distinguishing morphological properties. Over 250 morphological properties were calculated for each cell and used to construct a high dimensional feature space. Many dimensions in which cells from clusters A and B occupy significantly different ranges of values were found. Each dimension was then ranked by the Jenson-Shannon Distance (JSD) between the two clusters in the given dimension and the spearman correlation coefficient between the top ranking dimension and all other dimensions was calculated. In particular, we found TextureSumVariance5 a measurement of the local spatial variance (utilizing a co-occurrence matrix with a scale of 5 pixels see CellProfiler Documentation) of the image to be the top ranking dimension. SkeletonEndpoints, the number of endpoints in the skeleton of the cell mask was found to be poorly correlated with TextureSumVariance5 while maintaining a high JSD (we note that cell area was significantly different between the clusters as well). When projected onto this two dimensional space, clusters A and B were almost separable (Fig. 5h). It is clear that the high density biased cluster A contains cells with a “rougher” texture and more skeleton endpoints indicating a more uneven cell boundary. Thus the application of Self-Label ConvNet revealed the presence of distinct morphological phenotypes of potential biological significance.

## Discussion

The application of ConvNets to cell type segmentation and classification promises to improve the efficiency and accuracy of image analysis in many cell biology experiments. Challenges remain in improving the usability and generalizability of these methods. Protocols which do not require dedicated experiments or imaging will decrease the barrier-to-entry for labs new to these methods. The utilization of images taken on benchtop microscopes directly from cell culture flasks will make the use of the techniques presented more approachable.

For cell type identification, we found that cell morphological heterogeneity across flasks creates hurdles for network generalizability. However, we showed that when incorporating multiple flasks with multiple cell densities, good generalizability of the trained network is achievable. Mixed-population classification between two cell types was successfully performed, indicating the proposed ConvNet architecture is able to generalize to realistic experimental conditions. This may be especially useful when analyzing experiments with dynamic cell phenotype transformations including stem cell differentiation.

To gain a deeper understanding of morphological space identified through the use of our classification procedure, we developed an unsupervised morphological phenotyping technique called Self-Label Clustering (SLC), This method was shown to be able to identify unique morphological clusters that are cell density dependent. Taken together, the ConvNet cell morphology classification platform described in this work is a simple tool for biologists performing daily identification of different cell types or stages in cell differentiation. This method, together with other ML methods for image analysis and data quantification, could potentially transform the cell laboratory workflow and discover new biologically significant phenomena at the single cell level.

## Materials and Methods

### Data acquisition and preparation

T75 cell culture flasks of two cell lines, HEK-293A and HT1080, were seeded with a variable number of cells within a range achieving single-cell densities following typical cell passaging protocol. For cell detachment and passage, 0.25% trypsin (ThermoFisher Scientific) was applied to cells after washing with dPBS (ThermoFisher Scientific) twice. Cells were incubated for 5 minutes to allow for detachment. For experiments investigating density variation, low density flasks were seeded with 0.1 million cells and high density flasks were seeded with 0.5 millions cells counted by hemacytometer. Cells were allowed to adhere for 24hrs in flask before being imaged. For experiments investigating mixed-population cell culture, HEK-293A and HT1080 cells from two separate flasks were detached individually and mixed together in a 15 mL conical vial. Mixed cells were then seeded into a new flask (single cell density). The mixed-population flask was then incubated overnight for 24 hrs before being imaged. The HT1080 cell line utilized in the mixed-population studies was labelled with a fluorescent H2B-mCherry tag in the nuclei in order to provide a “ground truth” comparison with the unlabeled HEK-293A cells. The mixed-population test was conducted based on the pretrained model trained with multiple controlled and uncontrolled densities.

Brightfield images (1216 × 1616 pixels) of cells in a typical cell culture flask (Fig. 1b) were captured at 10X magnification with 0.25 NA (Numerical Aperture) on an OLYMPUS IX50 microscope, a standard benchtop model (Fig. 1a) equipped with Infinity Analyze (Lumenera Cor.), a software package used to operate the camera. All tests were imaged with identical settings and preparation with the exception that in the completion of the mixed-population tests, a fluorescent image was additionally captured for each region in order to resolve the H2B nuclear label in the HT1080 population. The camera used was not monochromatic and the images were converted to grayscale to reduce dimensionality and improve training efficiency. Single cells were then manually cropped and centered within squares of 224 pixels in length. The cropped images were augmented via image rotation, rigid translation, and the addition of artificial background imitating substrate irregularities before normalization (Fig. S1a). For the final data preparation step, we conducted quantile normalization (Fig. 1c) of the image intensity distribution to correct for any variations in light intensity across or among the fields of view. Each single cell image was normalized to a reference distribution constructed from an arbitrarily selected single cell image. The number of training images acquired and prepared was in the order of magnitude of 1,000, though varied with each individual test.

### Author summary

It is often difficult to distinguish different types of mammalian cells growing in cell culture. To this end, many types of biomarkers, anti-bodies and genotyping methods have been developed to classify cells, often requiring cell fixation and therefore sacrificing rare but interesting cells. Here we report that deep learning convolutional neural networks can visually distinguish mammalian cells with high fidelity using low resolution benchtop microscopes without any external labeling. The method is rapid and robust, and with sufficient training data, can be consistently applied across different types of experiments. The method is also successful in classifying cells in mixed population conditions, and therefore is useful for cell sorting and understanding cell phenotypic dynamics, right in the cell culture flask.

## Neural Network Design

### Cell type classification convnet

A graphical representation of the cell type classification neural network designed in the present work via MATLAB 2018a (MathWorks, Inc.) inspired by the structure of AlexNet^1^ was displayed in (Fig. 1d). The convolutional neural network is composed of the image input layer of size 224 × 224 × 1, followed by six quadruplets of a convolutional layer, a rectified linear unit layer, a batch normalization layer, and an average pooling layer, as well as one fully connected and one softmax layer at the end. The first convolutional layer has 32 kernels each of size 11 × 11 pixels and the subsequent five each have 32 × 32 pixel kernels of size 5 × 5 pixels. Stochastic gradient descent^18^ was chosen as the learning algorithm and the learning rate was set to be fixed at 10^*−*4^. We observed a learning rate within 10^*−*3^ to 10^*−*4^ to be appropriate for the tests conducted in this work, and the application of learning rate decay did not have a meaningful impact. Mini-batch size was set to be approximately 5% of the size of the dataset in each test and the mini-batches were designed to be shuffled randomly every epoch throughout the training process to enhance model validation performance. For training/validation set separation, in each test 80% of the images were assigned as training data and 20% of the images were assigned as validation data randomly. This random process was repeated 10 times for each test and the averaged statistics were reported. The 95% confidence interval on the classification error is calculated using the/formula $\epsilon \pm c\times \sqrt{\frac{\epsilon (1-\epsilon )}{N}}$ where *E* is the classification error, *N* is the observation size of validation set, and *c* is the constant 1.96. The ConvNet training was performed utilizing GPU (NVIDIA GeForce GTX 1060 6 G) on system with processor Intel(R) Core(TM) i7-7700K CPU @ 4.20 GHz (8CPUs) and 16GB RAM memory.

### Self-label convnet

A graphical representation of the Self-Label ConvNet designed for cell morphologicalSelf-Label ConvNetSelf-Label ConvNet phenotype clustering within one cell type via MATLAB 2018a (MathWorks, Inc.) wasSelf-Label ConvNetSelf-Label ConvNet 389 displayed in (Fig. 5a). The number of cells in the ensemble was indicated by *N* (in this Self-Label ConvNet study *N* = 2208). *N* classes were constructed in Self-Label ConvNet in the final layer (Softmax classification) instead of two classes for the cell type classification, while other layers before the final layer remained unchanged from (Fig. 1d), the cell type classification ConvNet. Each class in Self-Label ConvNet represents the combination of a series of *m* images (in this study *m* = 50) generated out of the same cell image through the augmentation outlined in (Fig. S1), and each class was then assigned a unique label of (“1”,“2”, through “*N*”) indicating *N* categories of distinguished Self-Label ConvNet morphological phenotypes throughout the ensemble. The training data of Self-Label ConvNet was then composed of *N* × *m* single cell images, leading to a much heavier computational cost for neural network training with around 3 million iterations to Self-Label ConvNet achieve stable accuracy and loss (Fig. 2b). Once the Self-Label ConvNet was successfully trained to nearly 100% accuracy, the pooled activations of the last convolutional layer of the ConvNet were investigated (see Results, (Fig. 5c,d).

## Expert Classification

To evaluate neural network performance and to additionally investigate similarities/contrasts between human and network feature identification, an expert classification survey was distributed to 20 individuals (Fig. S3). Four parts were included in the survey: 1. Within-flask pair classification between HT1080 and HEK-293A cells (Fig. 2b), 2. Classification between two flasks of a single cell type (cross-flask pair identification) (Fig. 2f), 3. Classification of two cell types when including multiple flask-pairs with uncontrolled density as training data and a novel flask-pair as validation data(Fig. 3e), and 4. multi-cell frame classification of two cell types in a single flask pair (Fig. 3f). Illustrated in (Fig. S3), experts were given 40 labeled images of cell type A and 40 labeled images of cell type B as preparation on one side of the survey paper (Fig. S3, top). They were then asked to classify 20 new unlabeled cells as either type A or type B on the survey paper (Fig. S3, bottom) in parts 1 through 3. In part 4, 40 multi-cell images were given for each cell type as labeled data and 20 new images were given as unlabeled data for expert classification. The performance of expert classification tasks was evaluated by the distribution of accuracy achieved by each individual expert and also average accuracy and standard deviation of all experts’ performances shown in (Figs 2b,f and 3e,f) for the 4 designed tasks respectively.

## CellProfiler

CellProfiler is a free, open-source image analysis software aiding users in image segmentation and the calculation of morphological features^19,20^ as well as subsequent statistical inference through CellProfiler Analyst^21^. CellProfiler was utilized in this study to provide connections between a standard morphological feature space constructed using a rigorous, well established pipeline and the clusters generated by the Self-Label ConvNet defined only by the novel, uncharacterized features learned by the neural network. To establish the morphological features which differ between the clusters, we calculated the Jensen-Shannon Distance between the two clusters in each dimension. The Jensen-Shannon Distance (JSD) is the square root of the Jensen-Shannon Divergence, also known as the total divergence to the average or the information radius (IRad), which itself is based on the Kullback-Leibler Divergence. The JSD between two (or more) probability distributions is a measure of the dissimilarity of those distributions. For two distributions, and using the natural logarithm, the JSD is bounded by 0 for identical distributions and ln(2) for dissimilar distributions. We found the clusters generated by the Self-Label ConvNet to have high JSD for many features calculated with CellProfiler. To select two dimensions on which to project the clusters to display their separability via morphological features, we went on to calculate the spearman correlation between each dimension and the dimension with the highest JSD (TextureSumVariance5). We then selected the second dimension (SkeletonEndpoints) to have high JSD but low correlation (Fig. S5e). Alternatively, pairs of dimensions may be more rigorously scored by measuring the separability of the clusters through a Support Vector Machine. We considered this method satisfactory for construction of the illustrative panel, (Fig. 5g).

## Supplementary information

Supplementary Information

## Data Availability

The data that support the findings of this study are available from the corresponding author upon reasonable request.

## Code Availability

All code used in the present work including but not limited to data preparation (single cell cropping, cell image augmentation and cell image normalization), ConvNet model construction, Self-Label Clustering package are available at https://github.com/sxslabjhu/CellTypeClassification.

## References

[1] Wang, X., Oxholm, G., Zhang, D. & Wang, Y. F. Multimodal transfer: A hierarchical deep convolutional neural network for fast artistic style transfer. *Proceedings of the IEEE Conference on Computer Vision and Pattern Recognition* (Vol. 2, No. 6, p. 7) (2017 Jul 1).

[2] Akram, S. U., Kannala, J., Eklund, L. & Heikkilä, J. Cell segmentation proposal network for microscopy image analysis. Deep Learning and Data Labeling for Medical Applications (pp. 21–29). Springer, Cham. (2016 Oct 21).

[3] Li, X., Li, W., Xu, X. & Hu, W. Cell classification using convolutional neural networks in medical hyperspectral imagery. Image, Vision and Computing (ICIVC), 2017 2nd International Conference on (pp. 501–504). IEEE.(2017 Jun 2).

[4] Xu Mengjia, Papageorgiou Dimitrios P., Abidi Sabia Z., Dao Ming, Zhao Hong, Karniadakis George Em (2017). A deep convolutional neural network for classification of red blood cells in sickle cell anemia. PLOS Computational Biology.

[5] Kihm, A., Kaestner, L., Wagner, C. & Quint, S. Classification of red blood cell shapes in flow using outlier tolerant machine learning. *PLoS computational biology*. **14**(6), e1006278.(2018 Jun 15). 10.1371/journal.pcbi.1006278 PMC6021115 29906283

[6] Ibrahim SF, van den Engh G (2007). Flow cytometry and cell sorting. InCell Separation.

[7] Smith Quinton, Rochman Nash, Carmo Ana Maria, Vig Dhruv, Chan Xin Yi, Sun Sean, Gerecht Sharon (2018). Cytoskeletal tension regulates mesodermal spatial organization and subsequent vascular fate. Proceedings of the National Academy of Sciences.

[8] Van Valen, D. A. *et al*. Deep learning automates the quantitative analysis of individual cells in live-cell imaging experiments. *PLoS computational biology*; **12**(11), e1005177 (2016 Nov 4). 10.1371/journal.pcbi.1005177 PMC5096676 27814364

[9] Shou, W., Bergstrom, C. T., Chakraborty, A. K. & Skinner, F. K. Theory, models and biology. *Elife*.; **4**:e07158 (2015 Jul 14). 10.7554/eLife.07158 PMC4501050 26173204

[10] Han, F. & Zhang, B. Characterizing Cell-Cell Interactions Induced Spatial Organization of Cell Phenotypes: Application to Density-Dependent Protein Nucleocytoplasmic Distribution. *Cell biochemistry and biophysics*; **65**(2):163–72 (2013 Mar 1). 10.1007/s12013-012-9412-8 22915253

[11] DEL CO´RDOBA-PEDREGOSA, M. C., Villalba, J. M., Gonzalez-Aragon, D., Bello, R. I & Alcain, F. J. Cellular density and cell type are the key factors in growth inhibition induced by 2, 5bis [1-aziridinyl]−1, 4 benzoquinone (DZQ). *Anticancer research*; **26**(5A), 3535–40 (2006 Sep 1). 17094478

[12] Singh, V. & Singh, S. M. Effect of high cell density on the growth properties of tumor cells: a role in tumor cytotoxicity of chemotherapeutic drugs. *Anti-cancer drugs*; **18**(10), 1123–32 (2007 Nov 1). 10.1097/CAD.0b013e3282ef5281 17893512

[13] Venugopal B, Mogha P, Dhawan J, Majumder A (2018). Cell density overrides the effect of substrate stiffness on human mesenchymal stem cells’ morphology and proliferation. Biomaterials science..

[14] Blatt, H. L, Rao, G. N. & Aquavella, J. V. Endothelial cell density in relation to morphology. *Investigative ophthalmology & visual science*; **18**(8):856–9 (1979 Aug 1). 378894

[15] Abdi, H. & Williams, L. J. Principal component analysis. *Wiley interdisciplinary reviews: computational statistics* (4), 433–59 (2010 Jul 2).

[16] Moghaddam, B. & Pentland, A. Probabilistic visual learning for object representation. *IEEE Transactions on pattern analysis and machine intelligence* (7), 696–710 (1997 Jul;19).

[17] Caron, M., Bojanowski, P., Joulin, A. & Douze, M. Deep clustering for unsupervised learning of visual features. *Proceedings of the European Conference on Computer Vision (ECCV)* (pp. 132–149) (2018).

[18] Bottou, L. Large-scale machine learning with stochastic gradient descent. *InProceedings of COMPSTAT’2010* (pp. 177–186). Physica-Verlag HD (2010).

[19] Carpenter, A. E. *et al*. CellProfiler: image analysis software for identifying and quantifying cell phenotypes. *Genome biology* (10), R100 (2006 Apr 7). 10.1186/gb-2006-7-10-r100 PMC1794559 17076895

[20] Kamentsky Lee, Jones Thouis R., Fraser Adam, Bray Mark-Anthony, Logan David J., Madden Katherine L., Ljosa Vebjorn, Rueden Curtis, Eliceiri Kevin W., Carpenter Anne E. (2011). Improved structure, function and compatibility for CellProfiler: modular high-throughput image analysis software. Bioinformatics.

[21] Jones Thouis R, Kang In, Wheeler Douglas B, Lindquist Robert A, Papallo Adam, Sabatini David M, Golland Polina, Carpenter Anne E (2008). CellProfiler Analyst: data exploration and analysis software for complex image-based screens. BMC Bioinformatics.

