# Supplementary material for: Cell Type Classification and Unsupervised Morphological Phenotyping From Low-Resolution Images Using Deep Learning
Source: Sci Rep. 2019 Sep 17;9:13467. doi: 10.1038/s41598-019-50010-9 (PMC6749053; doi:10.1038/s41598-019-50010-9)
Supplement: Supplementary file 1 — Supplementary Information [file 41598_2019_50010_MOESM1_ESM.pdf]

Cell Type Classification and Unsupervised Morphological Phenotyping From Low-Resolution Images Using Deep Learning: Supplementary Information

Kai Yao[*,1,2], Nash D Rochman[*,2], Sean X Sun[1,2,3]

1 Department of Mechanical Engineering, Johns Hopkins University, Baltimore, Maryland, United States of America
2 Institute for NanoBioTechnology, Johns Hopkins University, Baltimore, Maryland, United States of America
3 Physical Sciences in Oncology Center, Johns Hopkins University, Baltimore, Maryland, United States of America

* Authors Contributed Equally

Supplementary Figure Captions

Figure S1. Illustration of the single cell augmentation method. a. Single cell image augmentation through rotation and rigid translation and artificial background is added to increase robustness of the classification algorithm. b. Representative image files in the folder containing multiple augmentations of a single cell.

Figure S2. Convolutional activations (feature maps) as a product of ConvNet model training for the classification of two cell types. Representative unpooled convolutional activations of 6 convolutional layers for the two cell lines HEK-293A and HT1080. Each convolutional activation map contains 32 images corresponding to 32 kernels for each convolutional layer. The activations get blurrier visually over the depth of layers as the size of the activations gets smaller.

Figure S3. Expert classification survey page. The first expert task survey page displaying 40 representative images of each cell type to the experts and requiring the experts to classify novel cells.

Figure S4. Principle Component Analysis (PCA) outcome. PCA outcome for the HEK-293A cells from all cell densities. Two representative clusters were shown demonstrating the extraction of cell orientation through PCA.

Figure S5. Evaluation of the outcome of the Self Label Clustering (SLC) method. a. SLC proved robust to single cell augmentation outlined in Fig. S1. 9 augmentations were performed for 10 example cells respectively, and the LCA Feature Space for a cell (original and augmented version, 10 in total for one single cell) was shown within 10 consecutive rows. b. (top) Silhouette score curve of SLC over number of clusters chosen. A peak appears around k=11. (bottom) Clustering error of SLC indicated by the sum of the distances to cluster centers was plotted against the number of clusters chosen. At around k=11 a change of slope appeared. c. The Euclidean distance between every cell to 11 cluster centers. 2208 cells are plotted individually (rows) with their distances to the 11 cluster centers (columns) indicated by the purple color (the darker the larger). d. Representative cell images in two clusters of SLC (cluster \#3 and cluster \#10) for two densities: low density and high density. e. Identification of SkeletonEndpoints as Second Dimension of Interest. The Jensen-Shannon Distance (JSD) between the clusters in each dimension was plotted against the Spearman correlation between each dimension and TextureSumVariance5, the dimension with the highest JSD. SkeletonEndpoints (red circle) was identified as a dimension of interest with low correlation and high JSD.

Figure S1

a Single Cell Image Augmentation

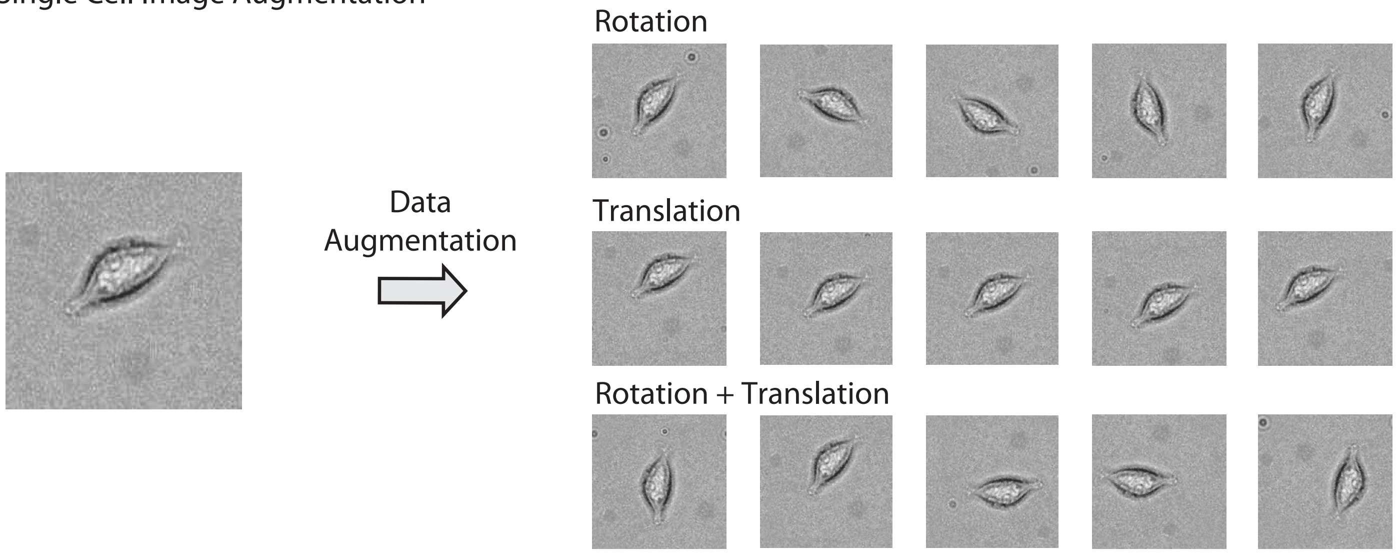

b

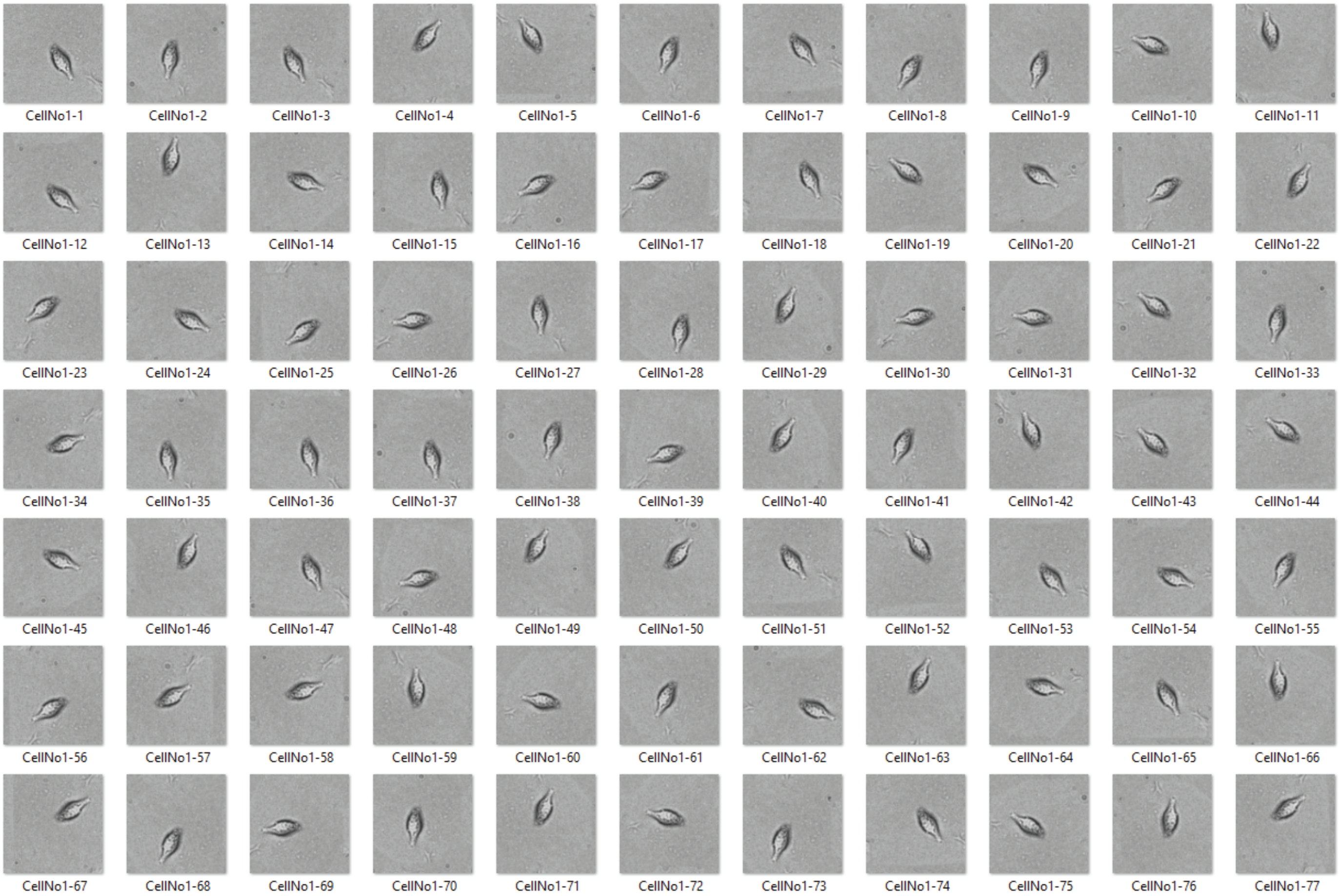

Figure S2

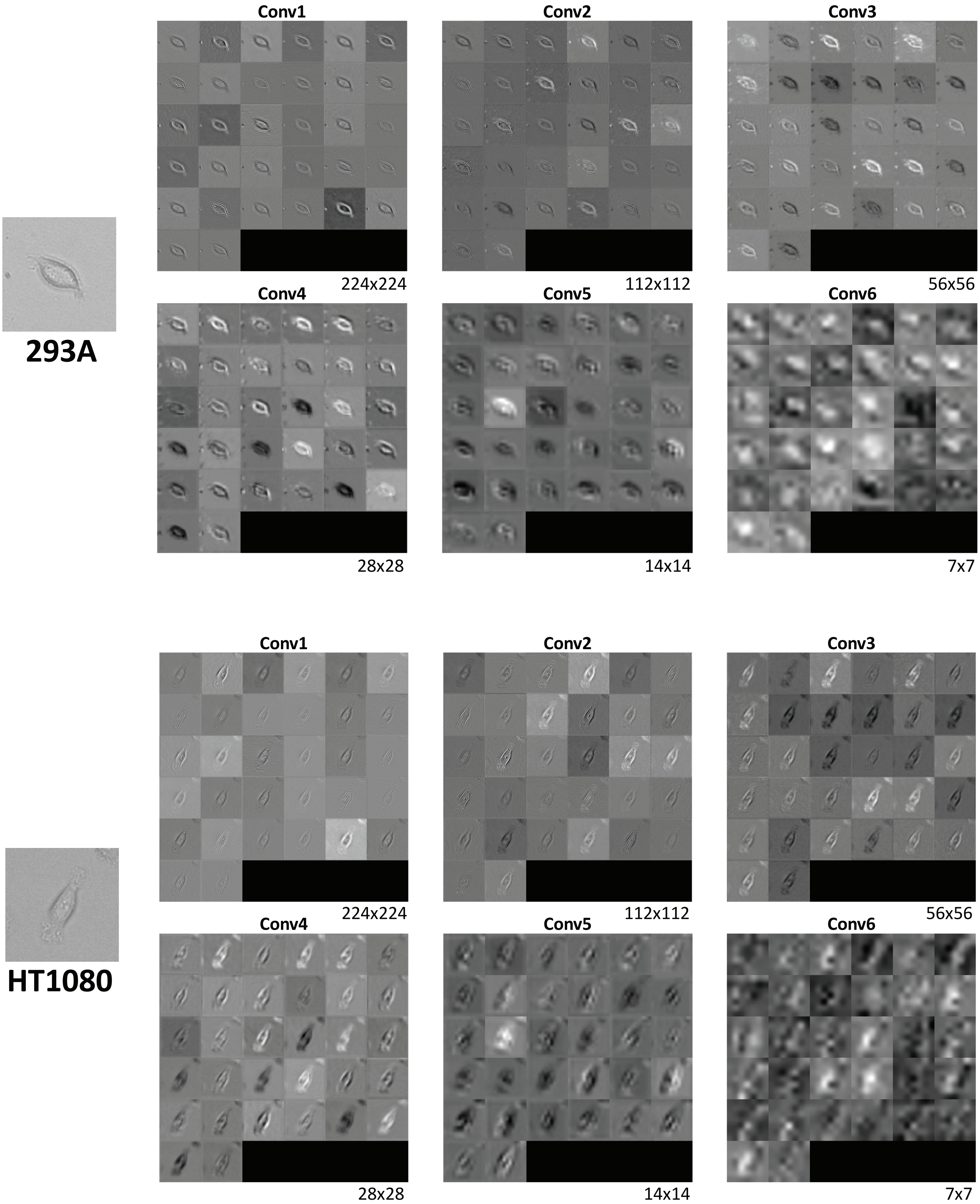
293A
Conv1
224x224
Conv2
112x112
Conv3
56x56
Conv4
28x28
Conv5
14x14
Conv6
7x7
HT1080
Conv1
224x224
Conv2
112x112
Conv3
56x56
Conv4
28x28
Conv5
14x14
Conv6
7x7

Figure S3

Expert Survey for Cell Classification by Humans

Example single cell images of two type of cells are given below, one is cell type A and the other one is cell type B

A

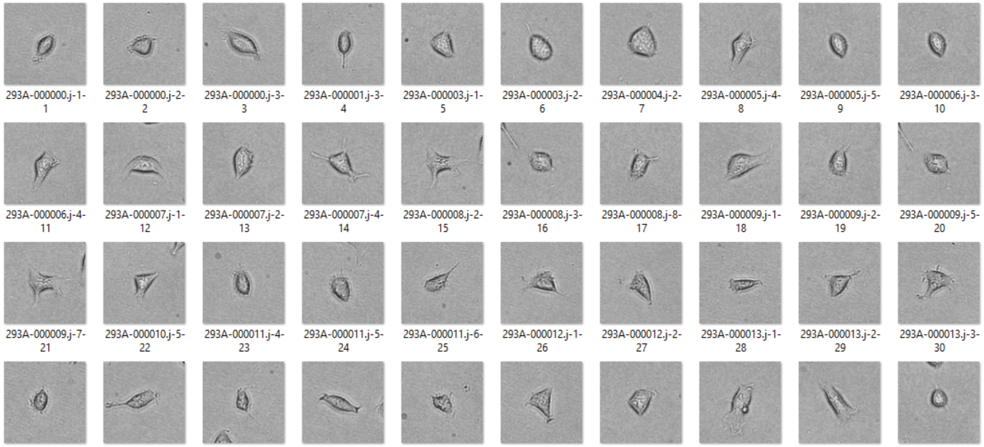

B

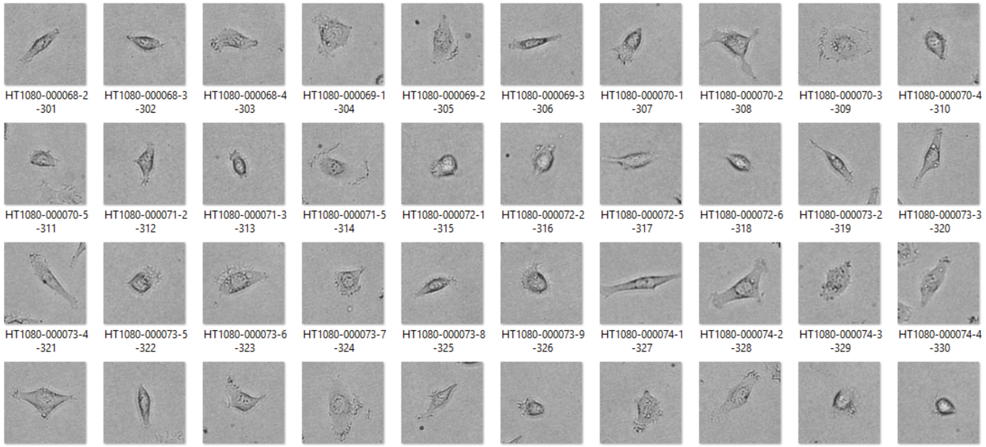

Please try to classify and label the following cells as A or B judging by your eyes

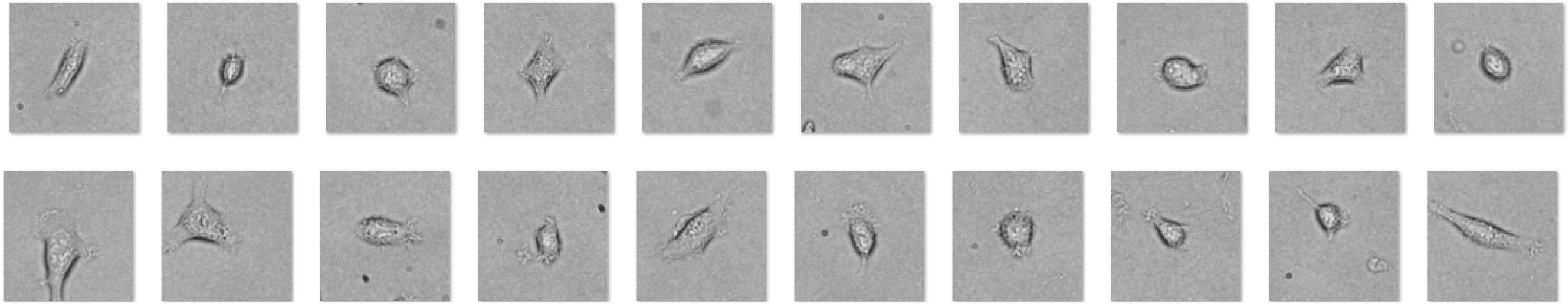

Figure S4

Cluster #1

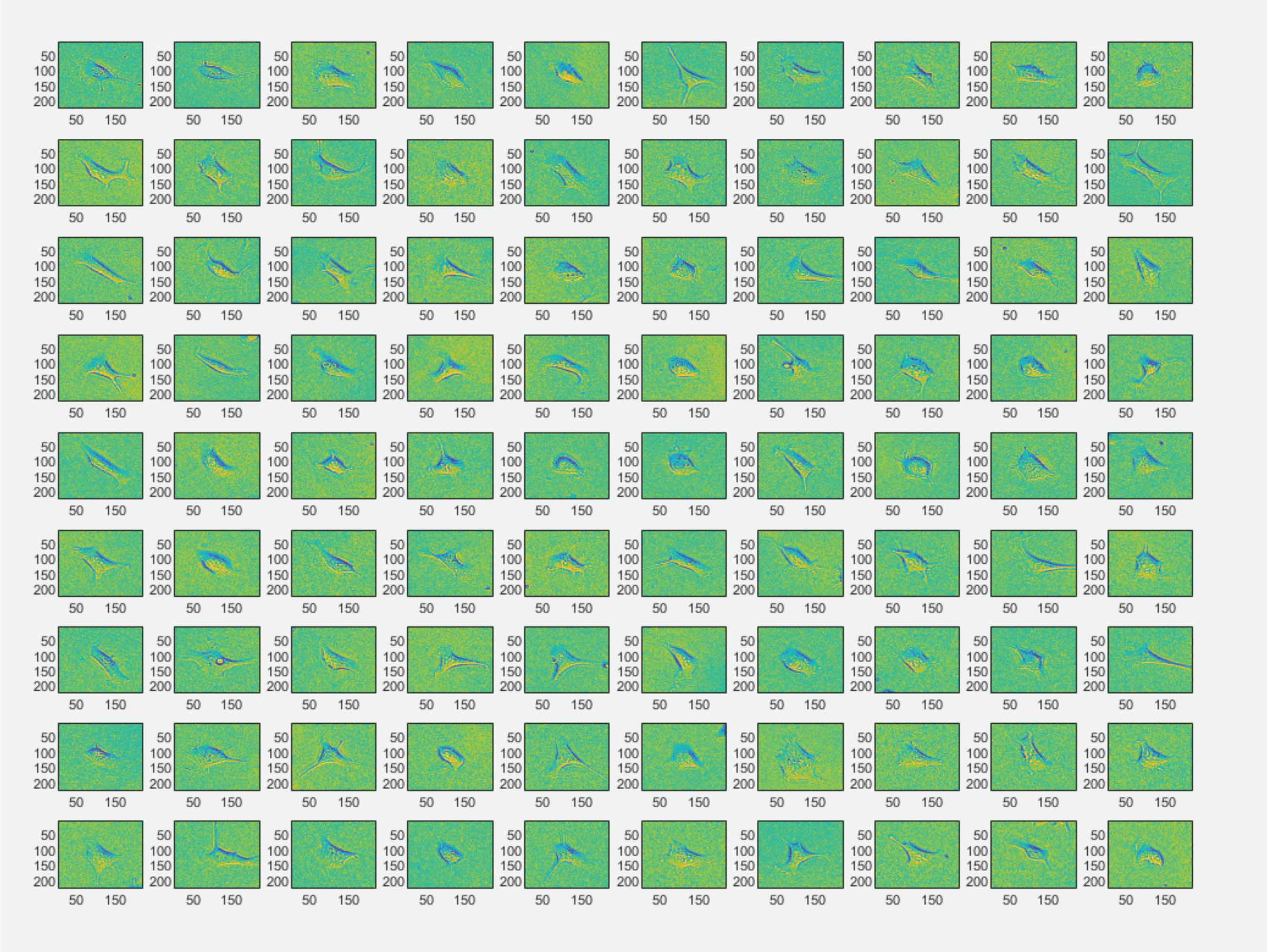

Cluster #2

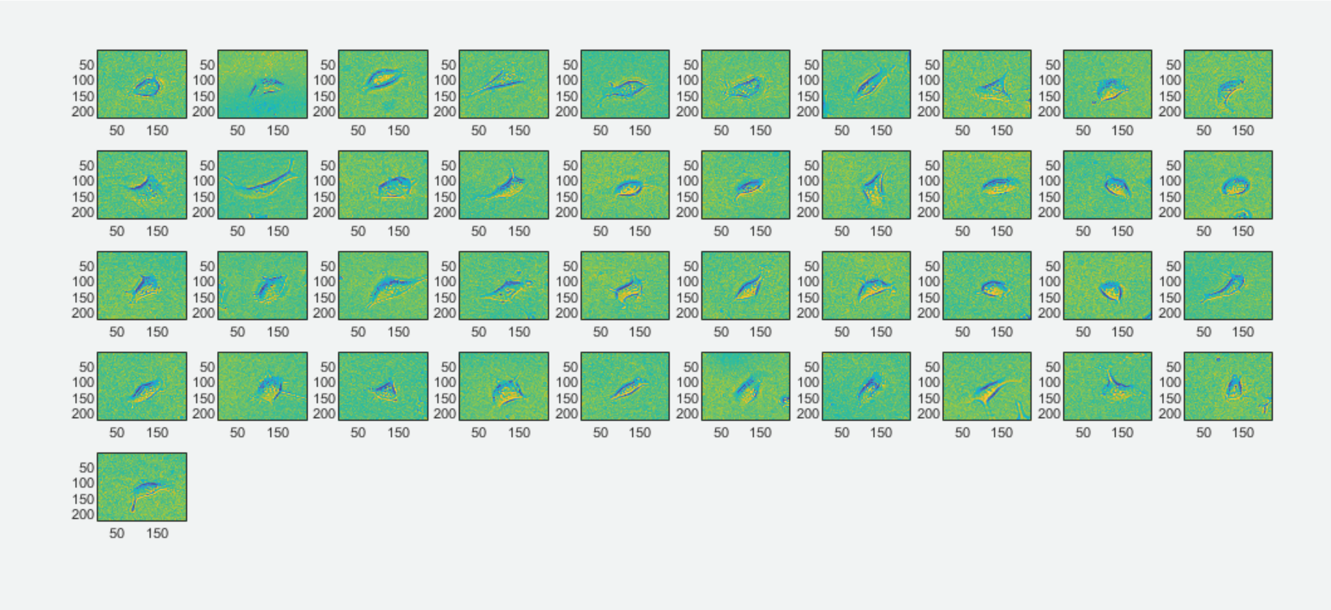

Figure S5

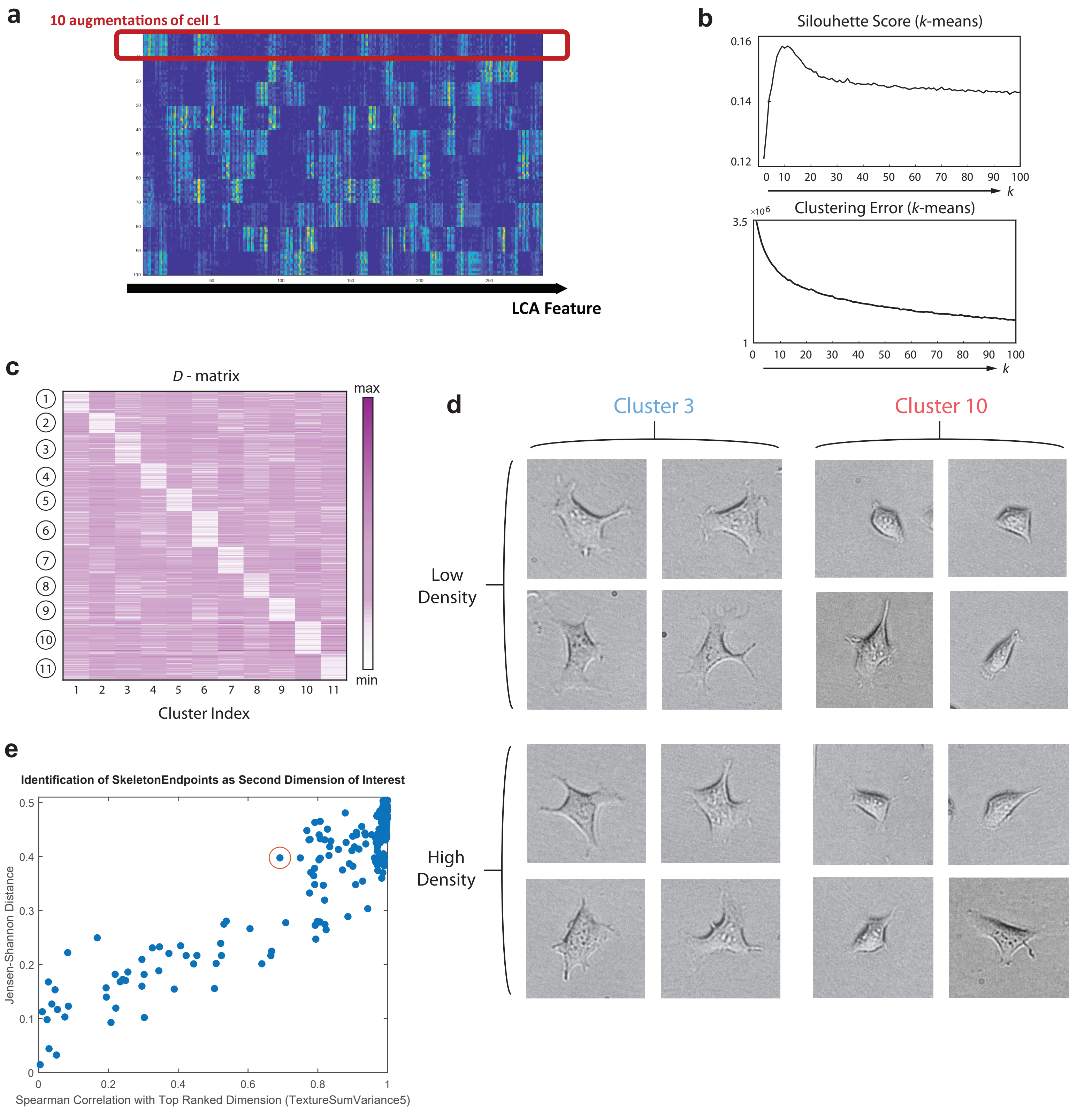
a
10 augmentations of cell 1
LCA Feature
b
Silouhette Score (k-means)
0.16
0.14
0.12
0 10 20 30 40 50 60 70 80 90 100
k
Clustering Error (k-means)
×10^6
3.5
1
0 10 20 30 40 50 60 70 80 90 100
k
c
D - matrix
max
min
1 2 3 4 5 6 7 8 9 10 11
Cluster Index
d
Cluster 3
Cluster 10
Low Density
High Density
e
Identification of SkeletonEndpoints as Second Dimension of Interest
Jensen-Shannon Distance
Spearman Correlation with Top Ranked Dimension (TextureSumVariance5)
